# Supplementary material for: Pharmaceutical Approach to Develop Novel Photosensitizer Nanoformulation: An Example of Design and Characterization Rationale of Chlorophyll α Derivative
Source: Pharmaceutics. 2024 Jan 18;16(1):126. doi: 10.3390/pharmaceutics16010126 (PMC10818748; doi:10.3390/pharmaceutics16010126)
Supplement: Supplementary file 1 [file pharmaceutics-16-00126-s001.zip › pharmaceutics-2731805-supplementary.pdf]

SUPPLEMENTARY MATERIALS

Pharmaceutical approach to develop novel photosensitizer nanoformulation: an example of design and characterization rationale of chlorophyll *a* derivative

Maria B. Sokol[1], Veronika A. Beganovskaya[1], Mariia R. Mollaeva[1], Nikita G. Yabbarov[1*], Margarita V. Chirkina[1], Dmitry V. Belykh[2], Olga M. Startseva[3], Anton E. Egorov[1], Alexey A. Kostyukov[1], Vladimir A. Kuzmin[1,4], Sergei M. Lomakin[1,5], Natalia G. Shilkina[5], Alexey V. Krivandin[1], Olga V. Shatalova[1], Margarita A. Gradova[5], Maxim A. Abakumov[6], Aleksey A. Nikitin[6], Varvara P. Maksimova[7], Kirill I. Kirsanov[7], Elena D. Nikolskaya[1,*]

[1] N. M. Emanuel Institute of Biochemical Physics of Russian Academy of Sciences, 119334 Moscow, Russia; mariyabsokol@gmail.com (M.S.); veronika.beganovckaya@bk.ru (V.B.); mollaevamariia@gmail.com (M.M.); yabbarovng@gmail.com (N.Y.); chir.marg@mail.ru (M.C.); ae.yegorov@gmail.com (A.E.); akostyukov@gmail.com (A.A.K.); vladimirkuzmin7@gmail.com (A.V.Ku.); lomakin@sky.chph.ras.ru (S.L.); a.krivandin@sky.chph.ras.ru (A.V.Kr.); shatalova@sky.chph.ras.ru (O.S.); elenanikolskaja@gmail.com (E.N.)

[2] Institute of Chemistry, Komi Scientific Center, Ural Division of the Russian Academy of Sciences, 167982 Syktyvkar, Russia; belykh-dv@mail.ru (D.B.)

[3] Pitirim Sorokin Syktyvkar State University, 167001 Syktyvkar, Russia; om_startseva@mail.ru (O.S.)

[4] National Research Nuclear University MEPhI, 115409 Moscow, Russia;

[5] N. N. Semenov Federal Research Center for Chemical Physics of Russian Academy of Sciences, 119991 Moscow, Russia; tashi05@list.ru (N.S.); m.a.gradova@gmail.com (M.G.)

[6] Laboratoty of Biomedical Nanomaterials, National University of Science and Technology (MISIS), 119049 Moscow, Russia; abakumov.ma@misis.ru (M.A.); nikitin.aa@misis.ru (A.N.)

[7] Blokhin National Medical Research Center of Oncology, 115478 Moscow, Russia; lavarvar@gmail.com (V.M.); kkirsanov85@yandex.ru (K.K.)

* Correspondence: elenanikolskaja@gmail.com, yabbarovng@gmail.com

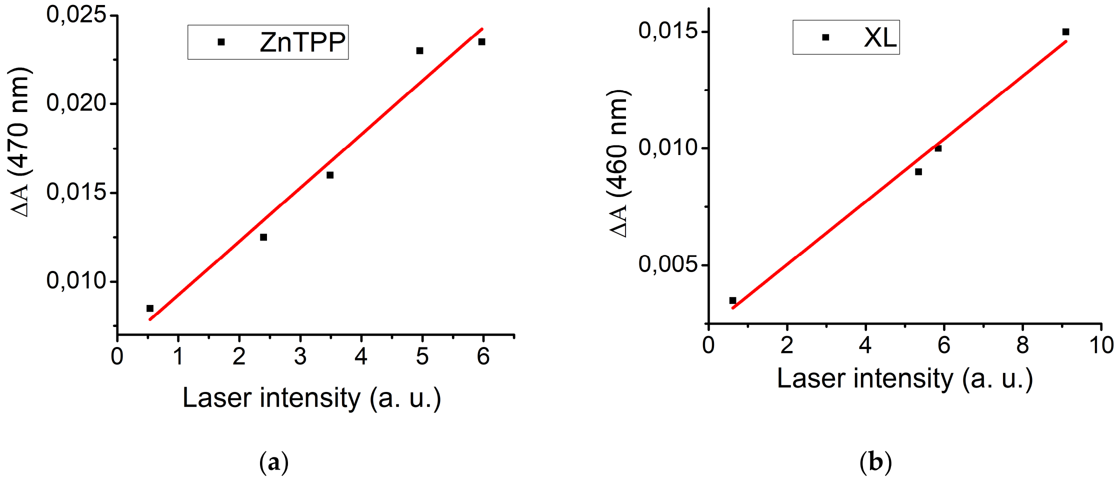

Figure S1. (a) Dependence between laser excitation energy and triplet state optical absorbance of ZnTPP (at 470 nm). (b) Dependence between laser excitation energy and triplet state optical absorbance of XL (at 460 nm). Excitation 419 nm.

Table S1. NP-XL long-term stability study data. Data shown as mean ± SD.

Time	Size, nm	PDI	ζ-potential, mV	DL, %	EE, %
0	222±10	0.243±0.012	-12.8±1.4	6.6±0.3	77±2
1 month	222±18	0.249±0.010	-12.0±1.7	6.6±0.2	77±3
3 month	227±13	0.245±0.010	-11.4±2.3	6.4±0.3	75±2
6 month	229±17	0.247±0.011	-11.5±2.0	6.3±0.3	75±2

Table S2. Data on biological activity predictions of XL with PASS-online service (http://way2drug.com/passonline/info.php). Accessed on 10.07.2022.

Pa	Pi	Cell line	Cell line full name	Tissue	Tumor type
0.608	0.012	HeLa	Cervical adenocarcinoma	Cervix	Adenocarcinoma
0.212	0.124	M19-MEL	Melanoma	Skin	Melanoma
0.312	0.236	NALM-6	Adult B acute lymphoblastic leukemia	Haematopoietic and lymphoid tissue	Leukemia

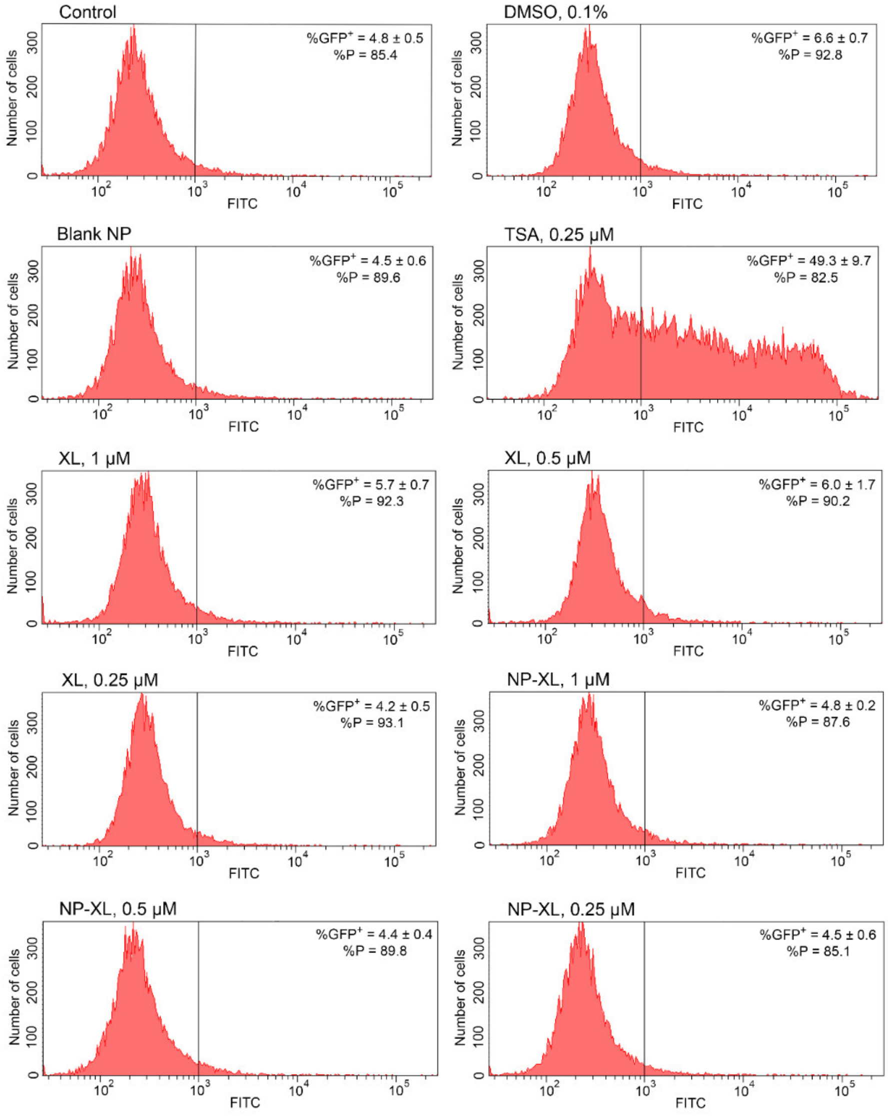

Figure S2. Flow cytometry analysis of reactivation of epigenetically repressed *GFP* gene expression in HeLa TI cells after exposure to XL, NP-XL and epigenetic modulator TSA. %GFP⁺, percentage of GFP-expressing cells; mean ± SD; %P, percentage of size- and granularity-matched live cells in the analyzed population.

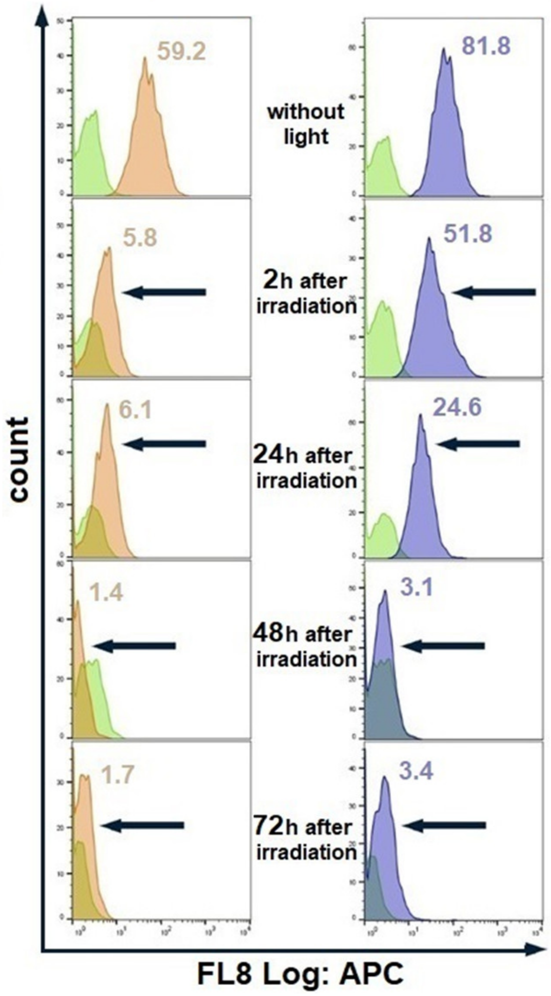

Figure S3. Flow cytometry analysis of HeLa cells treated with XL-8 (120 nM; orange) and XL-8-NPs (120 nM; blue) (not treated cells; green), irradiated and analyzed after 2 h, 24 h, 48 h, and 72 h. MFI of cells treated with XL-8 (orange numbers) and XL-8-NPs (blue numbers). Adapted from [1].

We tested XL-8-NPs photostability in comparison to XL-8 by evaluating its fluorescence intensity in HeLa cells after LED irradiation for 20 min. We demonstrated a time-dependent decrease in XL-8 and XL-8-NPs fluorescence intensity, as shown in Figure S3. However, we observed a higher fluorescence intensity of XL-8-NPs in comparison to XL-8 at 2 h and 24 h, which may be explained by XL-8 pro-longed release from NPs. According to these results, we concluded that XL-8 encapsulation in NPs preserves XL-8 photostability for 24 h and its safety.

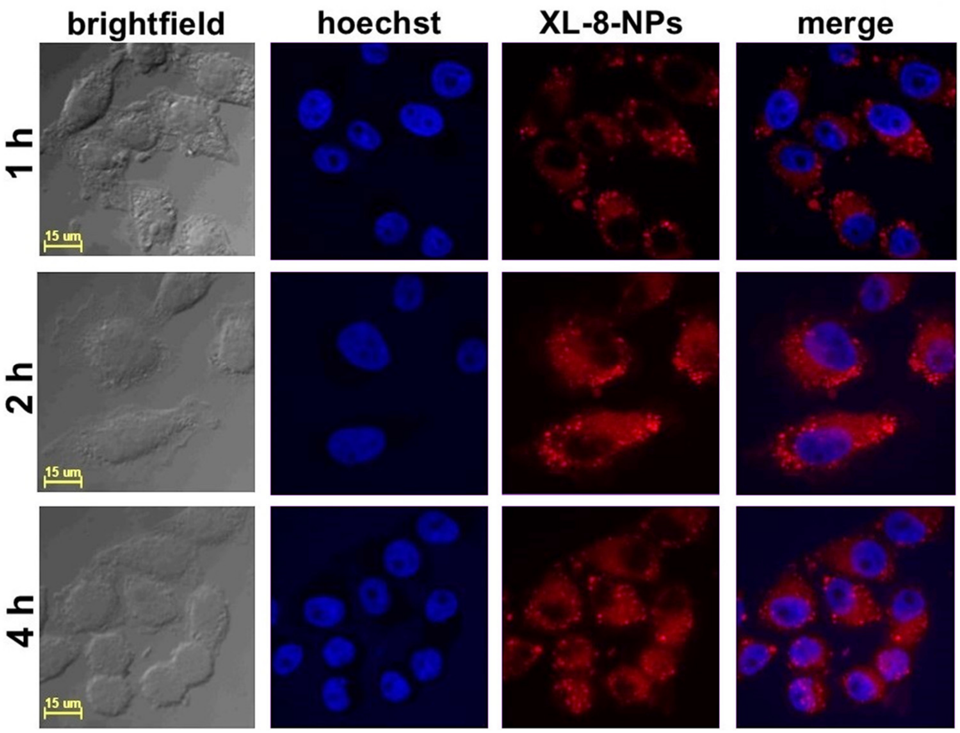

Figure S4. Confocal fluorescence images of HeLa cells treated with XL-8-NPs (120 nM) for 1, 2, and 4 h, with hoechst 33342. Adapted from [1].

We studied the intracellular accumulation efficacy of NPs (120 nM according XL-8) after 1, 2, and 4 h of incubation. The NPs demonstrated highly efficient accumulation in HeLa cells after 2 h of incubation (Figure S4).

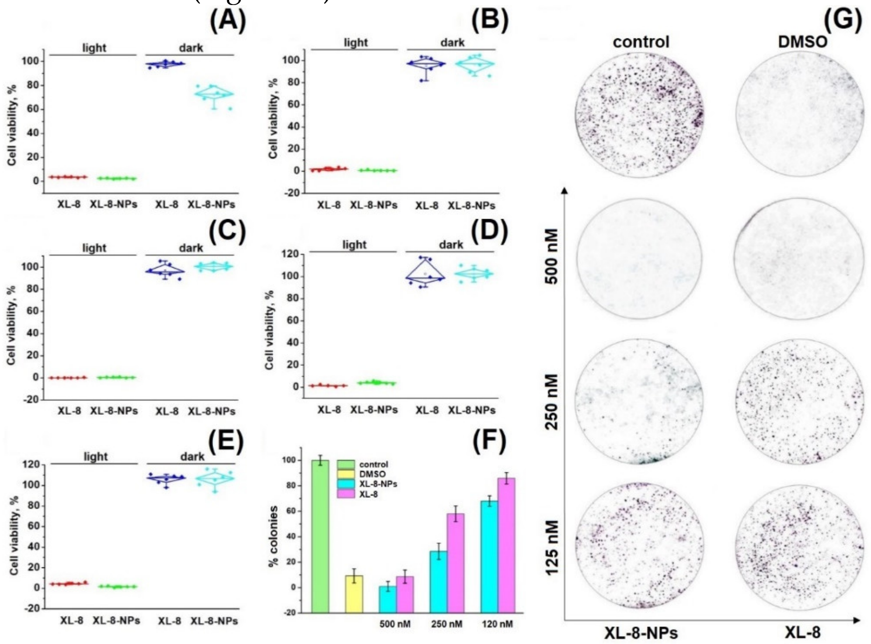

Figure S5. Photo- and cytotoxicity of XL-8 (500 nM) and XL-8-NPs (500 nM) analyzed by MTT assay. The percentage of cell viability was determined relative to viable control cells. SK-OV-3 cells (**A**), A549 cells (**B**), HeLa cells (**C**), MCF7 cells (**D**), and 4T1 cells (**E**) measured at 72 h after treatment. The quantification of colony formation rate in HeLa cells after treatment with 10% DMSO and XL-8- or XL-8-NPs-based PDT (**F**). Giemsa-stained plates of colony formation assay carried out on HeLa cells (**G**). Adapted from [1].

As shown in Figure S5 A–E, XL-8 and XL-8-NPs remained up to 80% of cell viability in the absence of light, indicating low dark toxicity. While using irradiation, XL-8 and XL-8-NPs exhibited a strong cytotoxic effect against all examined cells. HeLa cells demonstrated that they were the most sensitive to XL-8 and XL-8-NP.

Our data demonstrated also the efficiency of both XL-8- and XL-8-NPs to inhibit colony formation in a dose-dependent manner. Nevertheless, the growth rate inhibition ability of XL-8-NPs was considerably higher compared to free XL-8 (Figure S5 F,G). It is noteworthy that XL-8 and XL-8-NPs, at high concentrations, evoked a significant reduction in HeLa colony formation, while the control group formed large colonies.

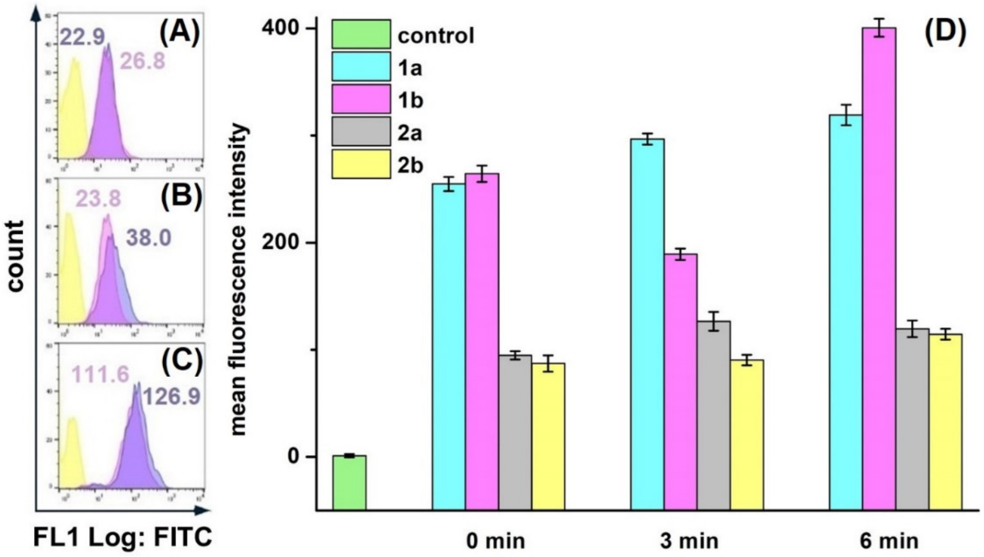

Figure S6. Different conditions of ROS registration in HeLa cells treated with XL-8 (100 nM; blue) and XL-8-NPs (100 nM; violet) (not treated cells; yellow) using DCFH-DA. Fluorescent dye added: after harvesting cells (**A**), irradiation (**B**), and PS treatment (**C**). MFI of cells treated with XL-8 (blue numbers) and XL-8-NPs (violet numbers). Evaluation of ROS (1) and superoxide anion (2) levels in HeLa cells, treated with XL-8 (a) and XL-8-NPs (b) (**D**). Adapted from [1].

We optimized conditions of intracellular ROS detection. Cells were stained with DCFH-DA after cell harvesting (Figure S6A), after irradiation; (Figure S6B), and before irradiation (Figure S6C). In XL-8 and XL-8-NPs treated samples, cells stained before irradiation and after harvesting displayed the similar fluorescence levels of DCF, comparable with un-stained cells. It is likely that the low MFI levels may be associated with the time-dependent process of DCFH-DA ester bond cleavage. Another important stage is cells harvesting by detachment with trypsin, followed by staining with DCFH-DA, which is a long-term process, possibly increasing additional cellular stress. In contrast, cells stained with DCFH-DA before irradiation facilitated the efficient ROS registration. Therefore, we applied the described staining methodology. HeLa cells displayed an intensive DCF green fluorescence after treatment with both formulations, evidencing the intracellular ROS formation. XL-8 demonstrated the strongest fluorescence intensity at 3 and 5 min after irradiation. The XL-8-NPs induced the higher signal after 6 min, evidencing the sustained XL-8 release and predominant particles accumulation in tumor cells (Figure S6D). DCF fluorescence live analysis (Figure S7 A,B) confirmed the most intensive ROS generation within the first 2.5 h after XL-8 and XL-8-NPs treatment and irradiation.

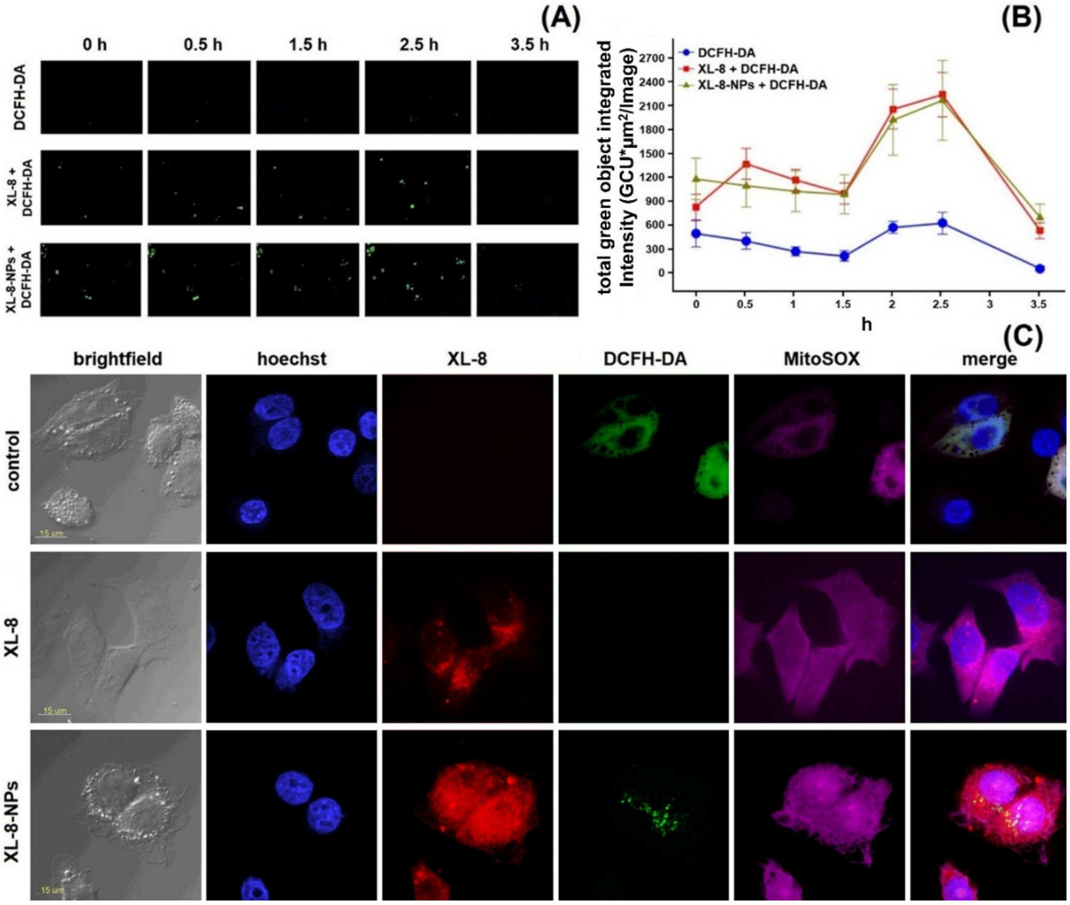

Figure S7. ROS formation kinetics in HeLa cells. Control cells were stained with DCFH-DA and irradiated. Two identical plates were treated and irradiated with XL-8 (100 nM) and XL-8-NPs (100 nM). Images were acquired every 30 min. Captured images from IncuCyte using a ×20 objective; 200 µm scale bars (**A**). At the end of analysis, an automatic real-time assessment was performed using IncuCyte, measured as green object integrated intensity for all cells stained green with DCFH-DA, allowing generating of graphical data (**B**). Fluorescent images of HeLa cells. Subcellular localization was investigated after treatment with XL-8 (100 nM) and XL-8-NPs (120 nM), hoechst 33,342, DCFH-DA, and MitoSox Red (**C**). Adapted from [1].

Considering the DCFH-DA low fluorescence specificity to particular ROS, we analyzed the XL-8-based vehicles' ability to induce mitochondrial superoxide formation using a MitoSox Red fluorescent probe. The fluorescence intensity of MitoSox Red displayed the similar profiles of superoxide anion and ROS formation. However, XL-8 induced stronger superoxide anion generation than XL-8-NPs (Figure S7 C). This effect may be explained by the lipophilic nature of XL-8, which promoted fast mitochondrial accumulation. The NPs (red signal) mainly displayed perinuclear localization (Figure S7 C); the pre-dominant dot-shaped fluorescence pattern indicated the endo-lysosomal accumulation, evidencing the endocytic nature of internalization, while sparse homogenous areas corresponded with the gradual XL-8 release from polymer matrix. We also revealed a partial overlapping of MitoSox Red, DCF, and XL-8 fluorescence, suggesting the PS accumulation in mitochondria. Relatively weak intranuclear MitoSox Red fluorescence may be explained by a time-dependent redistribution of dye. XL-8-NPs lacked co-localization with nuclei stained with hoechst 33342 but partially merged with DCFH-DA in the perinuclear area. XL-8 demonstrated weak intracellular entrapment but was able to stimulate a prominent MitoSox Red fluorescence. Interestingly, samples treated with XL-8 and XL-8-NPs al-most lacked DCFH-DA fluorescence compared to

control cells. These results demonstrated a high XL-8-NPs ability to internalize, redistribute along organelles, and, despite sustained release, stimulate oxidative stress at the same level as free XL-8 during the initial PDT phase.

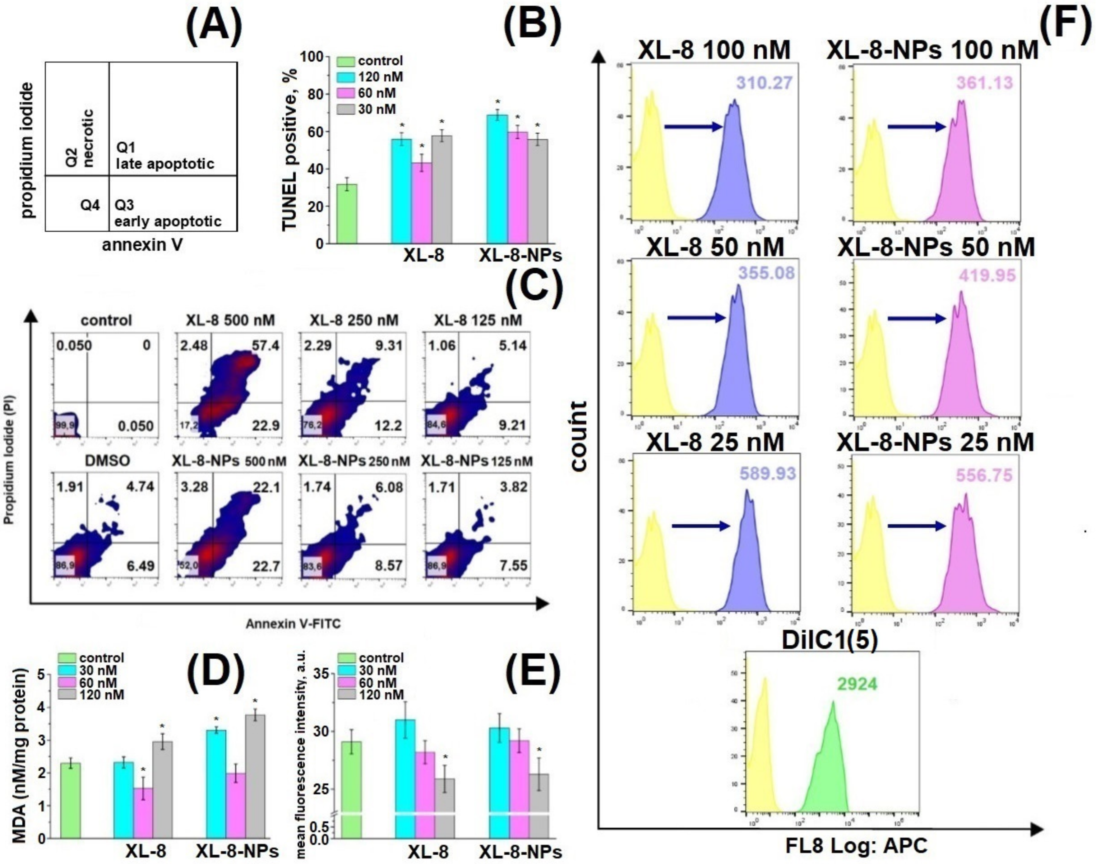

Figure S8. Schematic distribution of late apoptotic, early apoptotic, and necrotic populations in the corresponding quadrants (A). Percentage of TUNEL-positive HeLa cells treated with XL-8 and XL-8-NPs and irradiated. * $p < 0.05$ compared with the TUNEL treated group (control) (B). Apoptotic and necrotic HeLa cell populations after XL-8- or XL-8-NPs-based PDT. DMSO corresponds to 2% solution in DMEM (C). MDA analysis in HeLa cells. * $p < 0.05$ compared with the control group (TBA treated) (D). MFI of HeLa cells treated with XL-8 and XL-8-NPs, irradiated, and stained with o-phthaldialdehyde. * $p < 0.05$ compared with the control group (o-phthaldialdehyde treated cells) (E). Mitochondrial membrane potential (MMP, $\Delta\Psi m$) of HeLa cells treated with XL-8 and XL-8-NPs, irradiated (F). The results are shown as the mean ± S.D. (n = 3). Adapted from [1].

We applied annexin V-FITC/PI double staining to analyze cell death induced by XL-8 and XL-8-NPs (Figure S8 A). We observed a dose-dependent character of PDT-induced damage and formation of late and early apoptic populations (Figure S8 C). Up to 81.4% of cells were at different apoptosis phases after XL-8 treatment at a concentration of 500 nM compared to XL-8-NPs (43.7%); the late to early apoptotic populations ratio after XL-8 treatment was significantly higher than XL-8-NPs, but dose reductions of up to 250 nM and 125 nM mitigated this effect.

TUNEL assay revealed a dose-dependent manner of DNA fragmentation induced by ROS formation after XL-8- and XL-8-NPs-based PDT (Figure S8 B). XL-8-NPs (120 nM) treatment generated an increased number of TUNEL positive cells (75.99%) compared to XL-8 (55.89%) at the same concentration.

Changes in mitochondrial membrane potential (MMP) may evidence the organelle failure and lead to apoptosis. We applied a permeable lipophilic fluorescent cationic dye DiIC1(5) as a marker of mitochondrial membrane depolarization ($\Delta\Psi m$). Viable mitochondria with high membrane potential actively accumulated DiIC1(5), demonstrating a strong fluorescence, while in cells treated with XL-8

and XL-8-NPs mitochondria displayed dose-dependent fluorescence intensity decrease (Figure S8 F). The mitochondrial depolarization was a bit stronger in samples treated with 100 nM, 50 nM XL-8, and 25 nM XL-8-NPs, evidencing the oxidative stress and mitochondrial dysfunction.

Further, we analyzed the cytoplasmic oxidative stress markers to assess consequences of XL-8- and XL-8-NPs-based PDT. GSH supports intracellular RedOx homeostasis. PDT-induced GSH depletion may imbalance this system and trigger apoptosis. O-phthaldialdehyde interacts with intracellular GSH, forming fluorescent derivative. Both XL-8 and XL-8-NPs decreased GSH in a dose-dependent manner and displayed similar activity (Figure S8 E).

Along with GSH, we analyzed the level of malondialdehyde (MDA), one of the most prevalent and mutagenic lipid peroxidation products. We determined the MDA concentration in HeLa cells after XL-8- and XL-8-NPs-based PDT. Both formulations induced lipid peroxidation at high concentrations, but XL-8-NPs revealed a significantly higher activity (Figure S8 D). It is likely that the endocytic method of XL-8-NPs internalization, utilizing a lot of membrane components, promoted lipid peroxidation in contrast with free XL-8. Interestingly, XL-8 and XL-8-NPs at 60 nM decreased MDA levels to the reference value, which may be explained by the induction of proliferation under the mild oxidative conditions and activation of lipid synthesis.

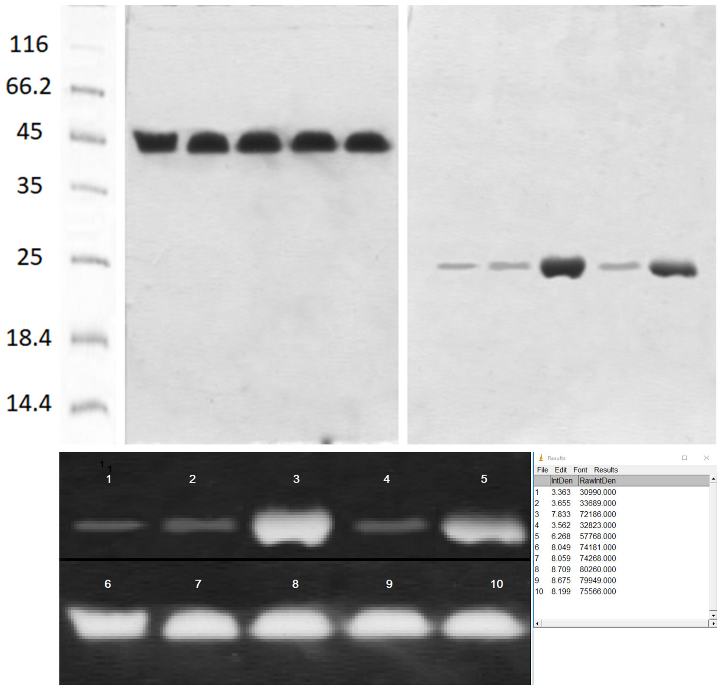

Figure S9. Western blot analysis of marker of apoptosis (cleaved PARP) in irradiated or non-irradiated MDA-MB-231 cells treated with XL (100 nM) or NP–XL (100 nM) (the cells were harvested 3 h after treatment).

Summary

We analyzed the phototoxicity of XL-8 and XL-8-NPs against SK-OV-3, HeLa, A549, MCF-7, and 4T1 cell lines, where HeLa cells displayed prominent sensitivity. It was shown that XL-8 entrapment in PLGA matrix preserved PS activity. Colony formation assay revealed an enhanced XL-8-NPs antiproliferative activity over XL-8. According to CLSM data, XL-8-NPs prevalently distributed along the cytoplasm, especially in the perinuclear area, and colocalized partially with mitochondria, which agreed with the report by Li et al., which described the Ce6 specificity to mitochondria. Interestingly, XL-8-NPs treated samples displayed a higher percentage of TUNEL-positive cells, which may be explained by the peculiarity of NPs internalization and perinuclear localization, promoting extensive DNA damage.

The main molecular mechanism of XL-8-induced PDT toxicity is based on oxidative stress. Both XL-8 and XL-8-NPs after irradiation triggered ROS generation during the first 6 min, while XL-8-NPs stimulated higher levels of ROS generation; at 3 h after PDT treatment, XL-8 and XL-8-NPs demonstrated the maximal levels of ROS formation. Recent reports described that photoirradiation

induces not only apoptosis but also ferroptosis, triggered by GSH depletion. Indeed, short time XL-8 and XL-8-NPs treatment induced GSH depletion, suggesting ferroptosis as a possible cell death pathway induced by PDT. MDA assay revealed an accumulation of lipid peroxidation products, which also indicated a ferroptosis. Furthermore, we clarified the mechanism of XL-8 and XL-8-NPs toxicity, investigating the involvement in apoptosis. Both vehicles induced apoptosis at low concentrations with insignificant difference, according to annexin V-FITC/PI staining. However, high XL-8 concentration more effectively induced the formation of late apoptic/necrotic population, which may be explained with the differences in internalization kinetics and interaction with membrane compartments. Next, we analyzed mitochondrial depolarization, involved in apoptosis, resulting in cytochrome c release and subsequent cell death. The XL-8 treated samples demonstrated a higher loss of MMP over XL-8-NPs treated sample, which may correlate with high lipophilicity and mitochondrial specificity of free PS.

These results revealed an activation of both apoptosis and ferroptosis pathways after XL-8 and XL-8-NPs-based PDT. Nevertheless, XL-8-NPs displayed more effective GSH depletion and lipid peroxidation, evidencing a stronger ferroptosis involvement, while free XL-8 provoked more prominent MMP loss and formation of late apoptic/necrotic populations, indicating that XL-8 induced apoptosis.

References

1. Mollaeva, M.R.; Nikolskaya, E.; Beganovskaya, V.; Sokol, M.; Chirkina, M.; Obydennyi, S.; Belykh, D.; Startseva, O.; Mollaev, M.D.; Yabbarov, N. Oxidative Damage Induced by Phototoxic Pheophorbide a 17-Diethylene Glycol Ester Encapsulated in PLGA Nanoparticles. Antioxidants 2021, 10, 1985.
